# Variability of Rayleigh and Moreland test results using anomaloscope in young adults without color vision disorders

**DOI:** 10.1371/journal.pone.0251903

**Published:** 2021-05-21

**Authors:** Jacek Zabel, Anna Przekoracka-Krawczyk, Jan Olszewski, Krzysztof Piotr Michalak

**Affiliations:** 1 Laboratory of Vision Science and Optometry, Faculty of Physics, Adam Mickiewicz University of Poznan, Poznan, Poland; 2 Laboratory of Bionics and Experimental Medical Biology, Department of Bionics and Bioimpendance, University of Medical Sciences, Poznan, Poland; 3 Laboratory of Vision and Neuroscience, NanoBioMedical Centre, Adam Mickiewicz University of Poznan, Poznań, Poland; University Eye Clinic, Ljubljana, SLOVENIA

## Abstract

**Aim:**

To validate the reference ranges proposed by the manufacturer of the Oculus HMC Anomaloscope MR for Rayleigh and Moreland tests in healthy young adults.

**Method:**

The manual Rayleigh (red-green) and the Moreland (blue-green) anomaloscope tests were performed on 90 healthy subjects (54 female, 36 male, 178 eyes) residing in Poland, aged between 18–45 years, and without color vision disorders (assessed with HRR test). The analyzed parameters for both the Rayleigh and the Moreland tests were as follows: the lower (R_1_/M_1_) and the upper (R_2_/M_2_) limits; the center (R_C_/M_C_) and the width (R_W_/M_W_) of the matching ranges.

**Results:**

The results of the Rayleigh test were similar to the values proposed in the anomaloscope user’s manual, however, with a small shift of R_C_ and R_2_ towards the red color. The double-peak distribution of R_2_ with a small second peak (approximately at R_2_ = 52) was mainly due to the measurements in male subjects (n_male_ = 8, n_female_ = 2), which suggests that this group might be diagnosed with subtle protanomaly. The results of the Moreland test showed a high M_W_ which did not correspond to the reference range described in the anomaloscope user’s manual. The observed significant correlations between R_1_ and M_1_ suggest that the M_1_ parameter seems to be the best indicator of blue vision quality.

**Conclusions:**

Oculus HMC Anomaloscope MR is a sensitive tool for detection of prot-deuteranomalies but the reference ranges for young adults require a certain adjustment towards the red color. The parameters obtained for the Moreland test varied significantly between the subjects and therefore the test should not be used as is to diagnose color vision deficits in the green-blue area (tritanomaly).

## Introduction

The history of methods used in diagnostics of color vision deficits dates back to the beginning of the 19^th^ century. However, the first conclusive tests became available at the beginning of the 20^th^ century. The tests available nowadays can be divided into screening (including Ishihara [1], HRR Pseudo Isochromatic Plates [2]) and diagnostic methods (such as Farnsworth 100 HueColor Vision Test or the anomaloscope [3, 4]). The general idea of the Anomaloscope is a comparison (matching) of two illuminated fields based on hue, saturation, and brightness [5].

Anomaloscopic measurement includes a comparison of the hues of two illuminated fields. For the red/green measurement, the reference field is illuminated with an adjustable proportion of red (666 nm) and green (549 nm) diodes which, depending on the proportion of red and green colored light, create the impression of green, lemon, yellow, orange or red color. The test field is illuminated with a yellow diode (589 nm) and the field’s brightness level can be adjusted by the test subject [6]. The proportion of red and green, which creates the impression that the color of the field has been equalized, is an indicator of the relative sensitivity of the eye to these colors. For example, subjects with protanomaly need the light from the red diode to be more intense in order to appreciate equalization of the test field color.

The first anomaloscope, created by Nagel [7], used a heating bulb and was powered by alternating electric current, which had an enormous impact on the measurement results. Richter [8] observed that color sensitivity was correlated to ambient temperature and the subject’s body temperature i.e. in high temperatures the results shifted towards the red area. This, in turn, could suggest a decrease in sensitivity to red color. The above observations were later confirmed by Jordan & Mollon [9] and Jagle et al. [5] who proved that voltage fluctuations in the power grid could significantly affect the intensity of the light emitted by the lamp and, as a consequence, affect the test results. In order to eliminate the measurement errors resulting from anomaloscope’s inaccuracy, Kintz designed a device with HP diodes used as light sources [8]. The diodes did not warm up and the power supply ensured a stable electric current. Subsequently, the anomaloscopes designed later were based on a similar solution. Currently, one of the commercially available new generation anomaloscopes is Oculus HMC Anomaloscope MR. The device is equipped with LEDs emitting light in three ranges (549, 589, 666 nm) which allows for examination of color perception in the red-green area (referred to as the Rayleigh test). Additional LEDs with light wavelengths of 436, 480, 490 nm facilitate a similar examination in the blue-green area (referred to as the Moreland test) [6]. Moreover, in the intervals between subsequent measurements, the device emits a neutralizing white light which enables the retina to regain a constant adaptation level.

The manufacturer provides reference ranges for both the Rayleigh and the Moreland tests [6] which allow practitioners to assign a patient to one of several groups of color vision disorders. The Rayleigh test is relatively well established and the narrow reference range specified by the manufacturer (from 34 to 46 units, where 0 corresponds to a fully green color and 73 units correspond to a fully red color) is deemed to be credible. In comparison, the Moreland test is less repeatable and its reference range (between 42 and 58 units, where 0 corresponds to a fully blue color and 100 units correspond to a fully green color), raises significantly more concern due to individual differences in macular pigmentation [10, 11] which may affect the perception of blue. The manufacturer has not provided clear references to the studies which were used as the basis for defining these reference ranges.

The majority of color vision deficits are related to the green-red area (protanomaly/protanopia or deuteranomaly/deuteranopia) while the congenital deficits in blue light perception (tritanomaly/tritanopia) are rare. Inherited tritan color vision deficiency has been reported to exhibit autosomal dominant inheritance pattern and to be caused by mutations in the S-cone opsin gene (OPN1SW) [12]. Six mutations associated with tritan color vision defects have been described in positions 56, 79, 190, 214, 264 and 283 of this protein. As for the R283Q mutation, the adaptive optics imaging suggested a progressive S-cone degeneration, which is correlated with progressively deteriorating blue–yellow color vision, thus constituting evidence that the inherited tritan color vision deficiency may progress with age [12].

On the other hand, there is an increasing number of studies that confirm acquired deficits in the blue-yellow axis. Papaconstantinou et al. showed the increasing Farnsworth-Munsell-100 test total error score in patients with primary open-angle glaucoma during a two year follow-up and observed that about 71% of patients showed the blue-yellow axis disturbance in the test. Five per cent of subjects showed changes in the red-green axis and 24% diagrams indicated to diffused changes [13, 14].

The aquired color vision deficiency in glaucoma was also evaluated by Tibber et al. [15] using the Rabin cone contrast test (RCTT). The researchers have confirmed a decrease in M- and S-cones RCTT, as compared to the control group, while the result for L-cones was close to significance level. The results for M- and S-cones were significantly correlated with the visual field loss score and macular ganglion cell/inner plexiform layer thickness in OCT.

In case of diabetes, Ong et al. [16] used the automated tritan contrast threshold test (TCT) to prove that the score was strongly correlated with the sight threating diabetic retinopathy score, assessed using slit lamp biomicroscopy and a 78D lens, even despite normal best corrected Snellen visual acuity result (BCVA).

The disturbances in color vision have been also reported in cases of viral infections, including HIV and cytomegalovirus retinitis. Kozak et al. [17] evidenced that early stage HIV-positive patients may exhibit S-cone/tritanopic abnormalities, measured by the S-cone luminance contrast sensitivity test (S-cone CS), despite normal fundus appearance and contrast visual acuity result.

Katz [18] analyzed a large cohort of patients with optic neuritis. The results of the FM-100 test showed that, during the acute phase of the disease, blue/yellow, red/green, and non-selective color defects occurred. Among patients with evident defects, the blue/yellow defects were more frequent than the red/green ones However, 6 months following acute corticosteroid treatment, their analyses showed that the red/green defects were more common than the blue/yellow defects. Similar results were presented by Menage et al. [19] who described a persistent deterioration in the FM-100 test in cases of demyelinating optic neuritis, even after successful recovery of visual acuity.

Moreover, transient tritanopia (reduction in sensitivity to purple stimuli following adaptation to a yellow display) has been reported during migraine episodes. The magnitude of this phenomenon was correlated with susceptibility to visually triggered migraines but not with migraine history, which suggests a transient retinal network disorder rather than cumulative retinal degeneration [15].

The purpose of this study was to evaluate whether the reference ranges provided by the manufacturer of Oculus HMC Anomaloscope MR are applicable to healthy young adults. The researchers focused mainly on the Moreland test as a potential future tool for detecting variations in crystalline lens transparency.

## Method

### Subject

The inclusion criteria were as follows:

- no color vision deficits detected in the HRR test (Richmond Products 4^th^ edition)—the test examines color vision in both the red–green and the blue areas;
- no ocular pathologies, this criterion was confirmed during ophthalmological examination performed by an experienced ophthalmologist (slit lamp with Volk lens and OCT with Maestro I, Topcon);
- no history of ocular pathology or ocular trauma;
- emmetropia or myopic refractive error up to 6 D or hyperopic refractive error up to 6 D and/or astigmatism of no more than 3 D;
- good distance and near best corrected visual acuity i.e. at least 0.1 logMAR in both eyes;
- no systemic diseases, such as diabetes, high blood pressure and/or endocrinological disorders.

Ninety subjects with normal color vision participated in the study (54 females and 36 males) and 178 eyes were examined (107 female, 71 male). Two eyes were excluded from the analysis due to certain issues with data recording. The mean age of the subjects was 31 years (range 18–45). The average absolute refractive error was 0.8 diopters (range from -3.5 to +2.0 diopters).

The study was approved by the local ethics committee of the Poznan University of Medical Sciences and the study protocol was designed in accordance with the declaration of Helsinki. A written informed consent was obtained from each participant.

### Procedure and analysis

The study was performed in a private eye care practice “Zabel” (located in Pila, Poland) in a room with low light intensity. Oculus HMC Anomaloscope MR (Oculus, Germany) was used for color sensitivity testing. Spectacle correction was used as necessary. The test was performed monocularly in a counterbalanced order and the subjects adapted to the light conditions for at least 10 minutes prior to the test. During the test, bright circular field (size of 2 degrees) divided into two halves was presented on the anomaloscope’s screen. The upper part of the circle served as the reference field and the lower part was the actual test field. In the manual mode, a researcher set variable values (i.e. color proportions) of the reference field and the observer (subject) had to adjust the brightness of the test field so as to achieve an equal color in both halves. In case the subjects were unable to match the colors, they were asked to report it to the researcher. In the Rayleigh test, the proportion of red and green light was adjustable while in the Moreland test the proportion of blue and green light was controlled. The test protocol used was in accordance with Oculus HMC Anomaloscope MR user’s manual. Detailed information concerning the procedure is provided in S1 Appendix.

The aim of the measurement was to define the limits of the range of color proportions in the test field within which the subjects were able to match the colors in both halves of the test.

The following set of four parameters was analyzed from the data obtained in both the Rayleigh and the Moreland test:

R_1_ –the lower limit of the matching range for the Rayleigh test, R_2_ –the upper limit of the matching range for the Rayleigh test, R_C_−central matching for the Rayleigh test, R_W_−the width of the matching range for the Rayleigh test (i.e. the difference between R_1_ and R_2_),

M_1_ –the lower limit of the matching range for the Moreland test, M_2_ –the upper limit of the matching range for the Moreland test, M_C_−central matching for the Moreland test, M_C_^R2<50^—central matching for the group of subjects where R_2_ was less than 50, M_C_^R2≥50^—central matching for the group of subject where R_2_ was higher than or equal to 50, M_W_−width of the matching range for the Moreland test (i.e. the differences between M_1_ and M_2_), M_W_^R2<50^—the width of the matching range for the group of subjects where R_2_ was less than 50, M_W_^R2≥50 –^the width of the matching range for the group of subjects where R_2_ was higher than or equal to 50.

The differences between male and female subjects were also analyzed. Then, due to 2-peak distribution of the R_2_ parameter, the differences between sexes were analyzed separately in the R_2_<50 group and the R_2_≥50 group. Finally, the correlations between Rayleigh and Moreland test parameters were analyzed and discussed.

Differences between left and right eyes were not observed and a strong correlation was observed between both eyes for individual subjects. Therefore, the analysis was performed on the mean values for both eyes for every parameter and each subject.

Statistica 13.1 software (StatSoft) was used for statistical analysis. Only one parameter (R_C_ in the Rayleigh test) showed a normal distribution and therefore non-parametric U Mann-Whitney test was used for data comparison. Statistical significance was achieved when the p-value was less than or equal to 0.05.

## Results

### Left and right eye comparison

The first step of the analysis included a comparison of left and right eyes of the participants. The comparison of results for the left vs. the right eye is presented in Table 1. It is noticeable that the results for every parameter were strongly correlated in the Spearman correlation and there was no difference between the eyes in the Mann-Whitney test. For that reason, the mean of the left and right eyes was calculated separately for every parameter and for each participant. Further analysis was performed using the mean values. In case of two subjects whose left eyes were excluded from the analysis, only the right eyes were included.

**Table 1:** Comparison of results for left and right eyes.

Parameter	Left eye mean	Right eye mean	Left eye median	Right eye median	Spearman correlation—R	Mann-Whitney test—p
M_1_	28.6±22.4	28.3±22.8	29.0	27.0	0.762	0.999
M_2_	84.0±6.1	85.1±6.9	85.0	86.0	0.504	0.080
M_C_	56.3±11.4	56.7±11.6	55.5	56.25	0.699	0.736
M_W_	55.4±23.7	56.7±24.4	56.0	58.5	0.743	0.735
R_1_	41.2±2.8	41.6±2.3	42.0	42.25	0.761	0.800
R_2_	47.4±2.2	47.5±2.5	47.5	47.0	0.683	0.759
R_C_	44.4±2.0	44.4±1.9	44.5	44.75	0.754	0.967
R_W_	6.1±3.0	6.0±3.0	5.5	5.5	0.755	0.767

No significant difference and a significant correlation between left and right eyes were observed for all of the analyzed parameters. R_1_ –the lower limit of the matching range for the Rayleigh test, R_2_ –the upper limit of the matching range for the Rayleigh test, R_C_−central matching for the Rayleigh test, R_W_−the width of the matching range for the Rayleigh test (the difference between R_1_ and R_2_) M_1_ –the lower limit of the matching range for the Moreland test, M_2_ –the upper limit of the matching range for the Moreland test, M_C_−the central matching for the Moreland test, M_W_−the width of the matching range for the Moreland test (the difference between M_1_ and M_2_), SD–the standard deviation of mean.

### Rayleigh test

**Fig 1:**
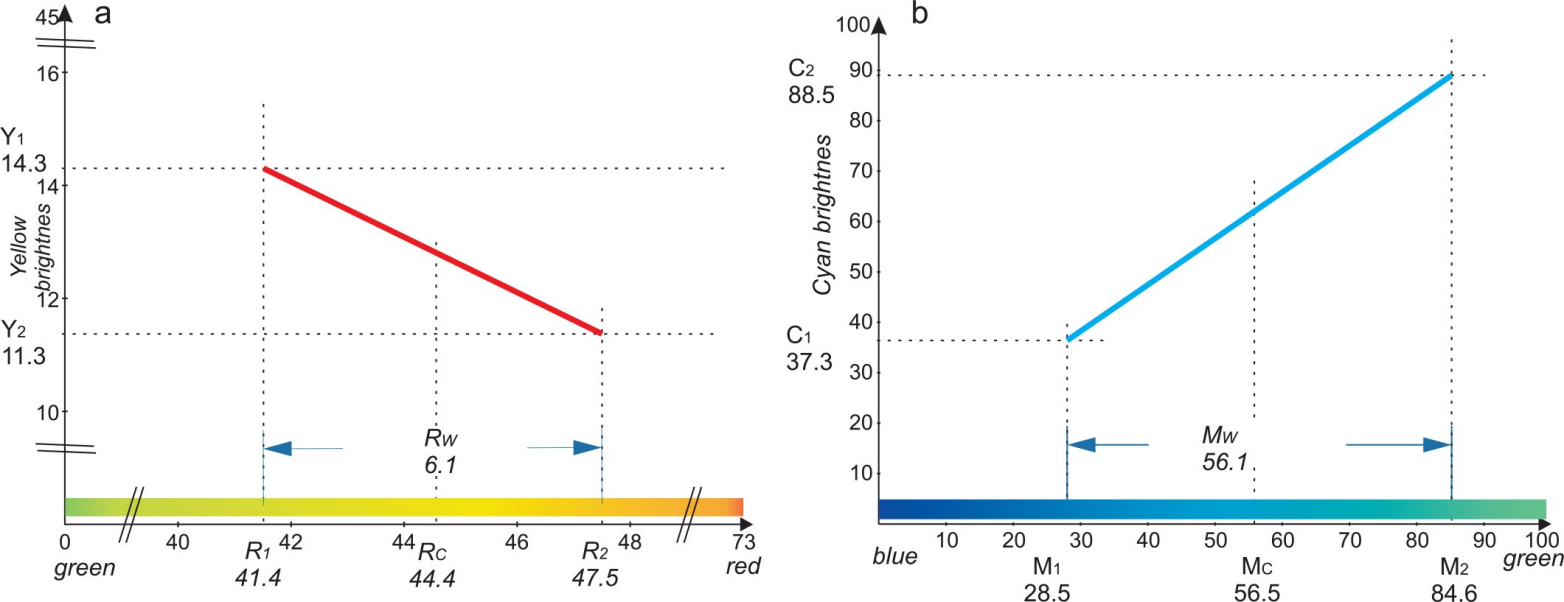
(a). A graphical presentation of the Rayleigh test parameters. Mean values of R_1_, R_2_, R_C_ and R_w_ parameters in the Rayleigh test are presented. Y_1_—the maximum brightness of yellow, Y_2_—the minimum brightness of yellow. (b) A graphical presentation of the Moreland test parameters. Mean values of M_1_, M_2_, M_C_ and M_w_ parameters in the Moreland test are shown. C_1_—the minimum brightness of cyan, C_2_—the maximum brightness of cyan.

The parameters describing the Rayleigh test observed in the study are presented in Table 2. The mean data obtained from the experiment is presented in Fig 1A and data distribution for this test is shown in Fig 3A. It is noticeable that the mean R_C_ for all the study participants was 44.4 and its value was slightly shifted towards the red for males (45.1) as compared to females (44.0, p = 0.022). The mean R_1_ value was 41.4 and was also slightly shifted towards red for males. However, it has not reached statistical significance level (m: 42.0, f: 41.0, p = 0.063). The mean R_2_ value was 47.7 and, again, the parameter’s values were significantly shifted towards the red for males as compared to females (m: 48.1, f: 47.1, p = 0.043). The mean matching range (R_W_) for the Rayleigh test was 6.1 (Fig 1A) and, as can be seen in Table 2, there was no significant difference between males and females (p = 0.715).

**Table 2:** Mean and median values obtained for all study subjects expressed in anomaloscope units, presented separately for males and females.

Detectability parameters	mean ± SD	Median	min—max	Males	Females	Males median	Females median	Mann-Whitney test (M vs F)
				mean ± SD	mean ± SD			
R_1_	41.4 ± 2.4	42.0	34.75–45.25	42.0 ± 1.9	41.0 ± 2.6	42.5	41.6	Z = -1.86, p = 0.063
R_2_	47.7± 2.2	47.25	43.25–55.5	48.1 ± 2.5	47.1 ± 1.9	48.0	47.25	Z = -2.02, p = 0.043*
R_C_	44.4 ± 1.8	44.5	39.87–49.25	45.1 ± 1.8	44.0 ± 1.7	45.1	44.1	Z = -2.23, p = 0.022*
R_W_	6.1 ± 2.9	5.5	1.75–16.75	6.0 ± 2.7	6.1 ± 3.0	5.6	5.5	Z = -0.36, p = 0.715
M_1_	28.5 ± 21.2	27.25	0–77	26.6 ± 19.7	29.8 ± 22.4	26.5	27.25	Z = 0.37, p = 0.708
M_2_	84.6 ± 5.3	85.5	61–92.5	83.6 ± 5.9	85.3 ± 4.8	84.25	85.5	Z = 1.59, p = 0.111
M_C_	56.6 ± 10.7	56.0	31.0–81.5	55,1 ± 9.8	57.5 ± 11.2	55.4	56.6	Z = 0.65, p = 0.514
M_W_	56.1 ± 22.46	57.5	8.5–90.5	57.0 ± 21.5	55.5 ± 23.3	58.8	57.5	Z = -0.24, p = 0.809

**Fig 2:**
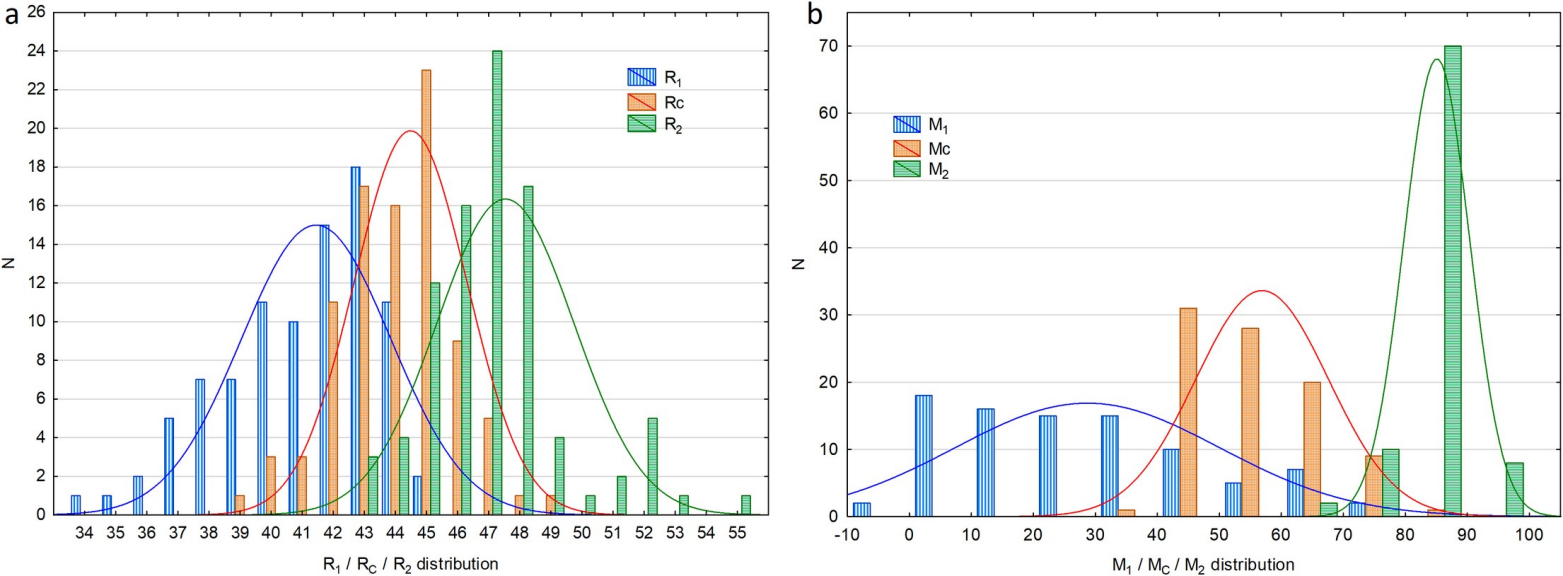
Distribution of a) R_1_, R_2_ and R_C;_ b) M_1_, M_2_ and M_C_; R_1_ –the lower limit of the matching range for the Rayleigh test, R_2_ –the upper limit of the matching range for the Rayleigh test, R_C_−central matching for the Rayleigh test, M_1_ –the lower limit of the matching range for the Moreland test, M_2_ –the upper limit of the matching range for the Moreland test, M_C_−central matching for the Moreland test; N–the number of eyes.

**Fig 3:**
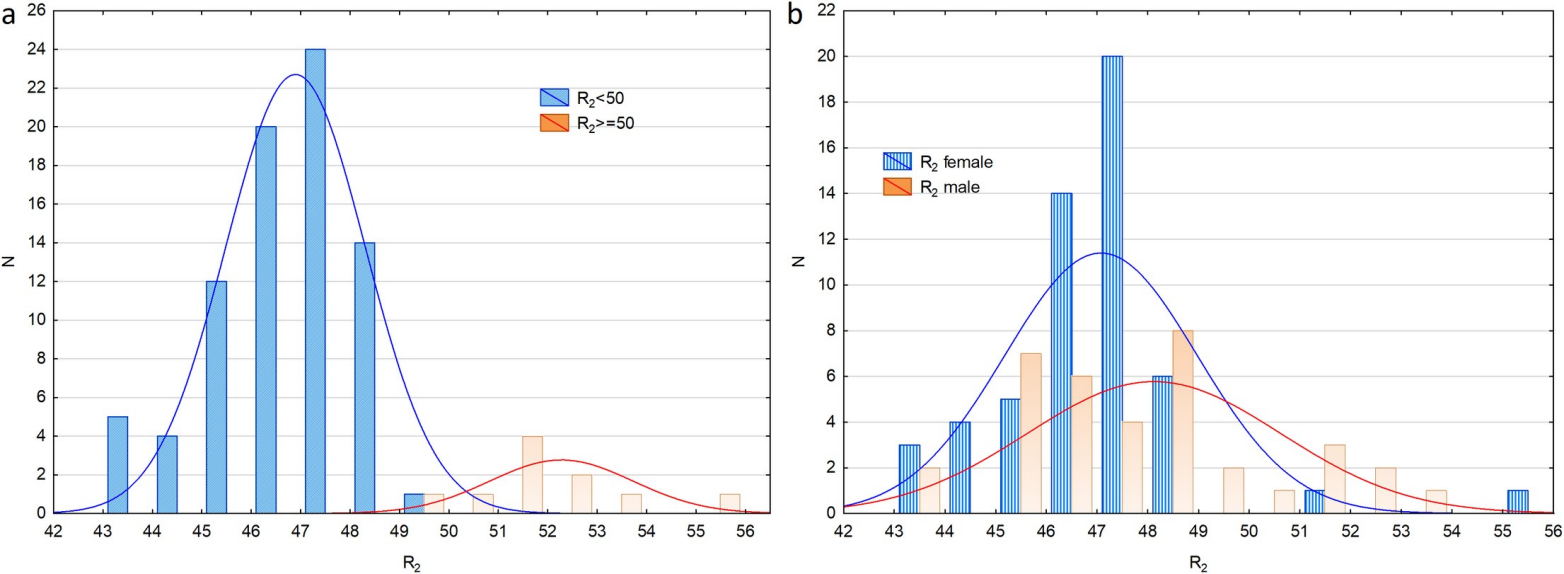
Distribution of R_2_ for the Rayleigh test. (a) Notice the two peaks: approximately at 47 and around the value of 52. (b) The distribution of R_2_ in the Rayleigh test is presented separately for female and male subjects.

It is noticeable in Figs 2A and 3 that the distribution of the R_2_ parameter shows two maxima (R_2max_ = 47 and 52) and a minimum of R_2min_ = 50. An additional analysis of the results for R_2_ ≥ 50 shows that the majority of such data was obtained from male subjects (8 subjects, see Fig 3A) with only two females in this group. It should be emphasized that the eight males constituted 22% (N_male_ = 36) of the examined male population while the two females (with R_2_ ≥ 50) constituted 3.7% of the study female population. Due to the above observation, an additional U Mann-Whitney test was performed which included only the eyes with R_2_<50 (N_male_ = 28, N_female_ = 52). This test has not shown any statistical significance between any of the Rayleigh test parameters for males and females (R_1_: p = 0.303; R_2_: p = 0.206; R_C_: p = 0.461, R_W_: p = 0.544).

### The Moreland test

The parameters describing the Moreland test are included in Table 2, the obtained mean data is presented in Fig 1B and data distribution is presented in Fig 2B. The mean M_C_ for all test subjects was 56.6 and the value was shifted slightly towards blue for males as compared to females but the difference was statistically insignificant (males: 55.1, females: 57.5, p = 0.514). The mean M_1_ was low (28.5) and the difference between the sexes was not significant (males: 26.6, females: 29.8, p = 0.708). M_2_ reached a value of 84.6 and, as above, the difference between males and females was insignificant (m: 83.6, f: 85.3, p = 0.111). The mean matching range (M_W_) for the Moreland test was high and amounted to 56.1 (Fig 1B). As Table 2 shows, the difference between males and females was statistically insignificant (p = 0.809).

### The subtle protanomaly group

As mentioned above, R_2_ has a two-peak distribution, with maxima approximately at 47 and 52, based on which the researchers assumed that the subjects in the R_2_ ≥ 50 subgroup showed a subtle protanomaly. Thus, additional analyses were performed separately for the group with R_2_ < 50 (further referred to as G^R2<50^) and for the group with R_2_ ≥ 50 (further G^R2≥50^). Table 3 shows the parameters measured separately in either of the groups and the statistical differences between the parameters (excluding R_2_). It is noticeable that two Moreland test parameters, i.e. M_1_, and M_W_, are statistically different in both groups. M_1_ is lower (15.3 vs. 30.2) and M_W_ is higher (70.4 vs. 54.3) in G^R2≥50^ as compared to G^R2<50^. Additionally, M_C_ is shifted towards blue (50.5 vs. 57.3) but it does not reach the significance level (p = 0.055). This suggests that the two groups represent different types of color vision and that color vision in the red-green axis modulates to some extent the ability to distinguish tones and hues in the blue-green axis.

**Table 3:** Comparison of 7 parameters (excluding R_2_) in the R_2_ < 50 group relative to the R_2_≥ 50 group (subtle protanopia).

Parameters:	R2<50 median	R2≥50 median	MW test	R2<50 Mean ± std	R2≥50 mean std
R_1_	41.9	43.25	Z = -0.79, p = 0.428	41.4 ± 2.3	41.7 ± 3.0
R_C_	44.3	47.25	Z = -4.42, p<0.001***	44.1 ± 1.6	47.1 ± 1.5
R_W_	5.0	8.6	Z = -3.91, p<0.001***	5.5 ± 2.2	10.3± 3.9
M_1_	28.5	14.0	Z = -2.01, p = 0.043^+^	30.2 ± 28.5	15.3 ± 12.2
M_2_	85.0	85.8	Z = -0.69, p = 0.490	84.5 ± 5.4	85.7 ± 4.4
M_C_	56.7	48.1	Z = 1.90, p = 0.055	57.3 ± 10.9	50.5 ± 6.1
M_W_	54.0	71.5	Z = -2.18, p = 0.028^+^	54.3 ± 22.7	70.4 ± 13.6

The key to symbols is the same as in Table 1. In the Moreland test, subjects with subtle protanomaly showed significantly different values of M_1_ and M_W_ and close to significant values of M_C_ (p = 0.055). The groups did not differ regarding the R_1_ parameter.

In the Rayleigh test, very high and significant differences between the groups were observed for parameters R_C_ and R_W_ (p < 0.001) but not for R_1_. The mean R_C_ was equal to 44.1 in G^R2<50^ and 47.1 in G^R2≥50^, respectively. The mean R_W_ was equal to 5.5 in G^R2<50^ and 10.3 in G^R2≥50^ group. The lack of a significant difference for R_1_ suggests that in cases of subtle protanomaly, the R_1_ parameter is not really meaningful and the increase in R_C_ and R_W_ is mainly due to R_2_ shift towards red.

The differences between males and females in G^R2<50^ and G^R2≥50^ groups were also analyzed. No difference was found between male and female subjects for all the parameters except for M_2_ in G^R2<50^ (males 82.9, females 85.3, p = 0.031). This observation supports the conclusion that the increased mean values of the Rayleigh test parameters between the sexes were mainly due to the male subjects from the subtle protanomaly group. In the G^R2≥50^ group, the R_1_ and R_W_ parameters differed significantly between male and female subjects. R_1_ was lower and R_W_ was higher for females. However, this difference must be analyzed with caution as only two females in this group were included in the analysis.

### Correlations between Rayleigh and Moreland tests

Additional correlations between the Rayleigh and the Moreland test parameters were analyzed, due to the significant intersubject variability of the Moreland test results and in order to allow for a better understanding of the factors impacting color vision in the blue-green range. The results of Spearman correlations between the analyzed parameters are presented in Table 4. Noticeably, R_2_ and R_C_ are poorly or not correlated with the Moreland test parameters. Similarly, M_2_ is poorly or not correlated with the parameters of the Rayleigh test. Six significant correlations ranging between R = 0.35 and 0.51 have been observed between R_1_/R_W_ and M_1_/M_W_/M_C_, respectively. These correlations are presented in Table 4.

**Table 4:** Spearman correlations between parameters of the Moreland and the Rayleigh tests.

Spearman correlations	R_1_	R_W_	R_C_	R_2_
M_1_	R = 0.383^**^	R = -0.481^***^	R = 0.103	R = -0.224^+^
M_W_	R = -0.371^**^	R = 0.514^***^	R = -0.073	R = 0.288*
M_C_	R = 0.350^**^	R = -0.424^***^	R = 0.098	R = -0.172
M_2_	R = -0.149	R = 0.317*	R = 0.021	R = 0.300*

The key to symbols is the same as in Table 1.

+: p<0.05

*: p<0.01

**: p<0.001

***: p<0.0001.

**Fig 4:**
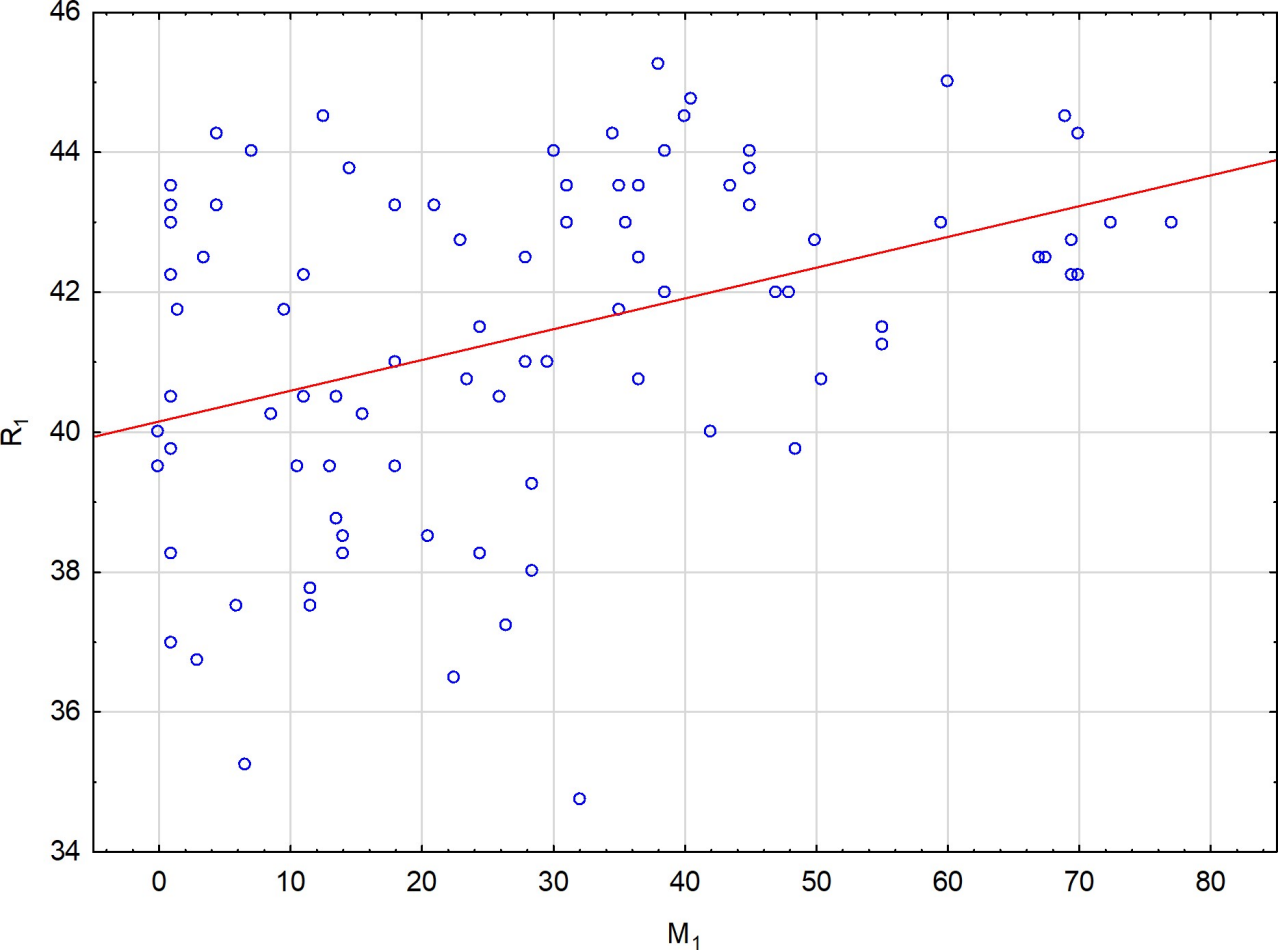
Regression between the lower limit of the matching range in the Rayleigh test (R_1_) and the lower limit of the matching range in the Moreland test (M_1_). R = 0.383, p<0.001.

Because R_2_ and M_2_ showed poor or no correlations, it may be concluded that M_C_ and M_W_ correlations are mainly due to M_1_ variability. Similarly, the correlations of R_C_ are related to R_1_ variability and therefore the relation between R_1_ and M_1_ seemed to be the most important one. The results are presented in detail in Fig 4 which shows that the correlation between R_1_ and M_1_ occurred mainly due to the lack of subjects with low R_1_ (wide R_W_) and high M_1_ (narrow M_W_).

The subjects who were found to have a narrow matching range in the Moreland test (M_1_≥40) also showed a high R_1_ and narrow R_W_ in the Rayleigh test. High M_1_ indicates a good ability to see blue. This ability was related to a better discrimination of the green-lemon-yellow hues in the Rayleigh test (high R_1_). As there were no subjects with low R_1_ and high M_1_, it may be argued that good hue discrimination in the red-green axis may be an important factor for good hue discrimination also in the blue-green axis. This relation may be due to the fact that red cones show sensitivity to blue light. Modulation of information from the blue vs green cones by information from the red cones may be an important factor in determining the tones and hues in the blue-green range. However, verification of this hypothesis will require further research.

## Discussion

The aim of the study was to determine the norms for both sexes for the Moreland test and to establish the common relations between Rayleigh and Moreland tests. A detailed discussion of the results obtained in this study is presented further in the text.

### Rayleigh test

The study was focused on the validation of the reference ranges for the Oculus HMC Anomaloscope MR in a non-presbyopic population of healthy young adults. All study participants were free of any systemic and ocular pathologies. None of them reported any problems with color vision and all subjects have passed the HRR color vision test.

The results obtained in the Rayleigh test differed slightly from those described by the authors of the anomaloscope user’s manual as the reference range for the Rayleigh test [6]. Assuming that the population norms are as follows: for R_1_ the norm is R_1norm_ = mean (R_1_)– 2 x SD of (R_1_) and for R_2_ it is R_2norm_ = mean (R_2_) + 2 x SD of R_2_, then the resulting values are R_1norm_ = 35.9 and R_2norm_ = 52.6. The range of reference values specified in the anomaloscope user’s manual is between 34 and 46 units (with a mean of c. 40 units) while the results obtained by the authors indicated that the range should be approximately 36–53 units (with a mean of c. 44 units).

Additionally, due to the differences between males and females [20, 21], the authors suggest that separate norms should be used for both sexes. The suggested norms for females are R_1norm-f_ = mean (R_1_^f^)– 2SD (R_1_^f^) = 41.0–2 x 2.8 = 35.4 and R_2norm-f_ = mean (R_2_^f^) + 2SD (R_2_^f^) = 47.1 + 2 x 2.1 = 51.3. Similarly, the suggested revised norms for males is R_1norm-m_ = mean (R_1_^m^)– 2SD (R_1_^m^) = 41.4–2 x 2.3 = 36.8 and R_2norm-m_ = mean (R_2_^m^) + 2SD (R_2_^m^) = 48.8 + 2 x 3.0 = 54.8.

Another finding is that the R_2_ parameter was most sensitive for detecting pathologies related to red color vision. The R_C_ and R_W_ parameters, which were also sensitive in detecting red color vision pathologies, depended solely on R_2_. Moreover, R_1_ was poorly correlated with R_2_ (r = 0,22) and did not differ significantly between the genders or the G^R2<50^/G^R2≥50^ groups.

Similarly, the variability of R_1_ should probably be treated as the most sensitive indicator of deuteranomaly. The presented results suggest that R_1_ and R_2_ are related to different aspects of color vision and should to be analyzed separately.

It should also be verified whether the group allegedly showing subtle protanomaly (G^R2≥50^) should be treated as being within normal limits or as an actual subtle pathology. It was shown that small changes in photopigment peak spectral sensitivity shifts were found even in the healthy population [22] and that not only significant defects but also subtle interpersonal differences in cones density are coded genetically [23]. Thus, it seems possible that congenital interpersonal changes in cones characteristics may account for the variation found in Rayleigh matching. Such individuals constitute about 20% of the male population while the reference norms are usually described as including 95% of the population. However, the measured red color vision results in this group were significantly poorer than those observed in females and in the remaining 80% of the male population. Thus, a differentiation of the male population into two sub-groups, i.e. one with a good red color vision and the other showing a slightly weaker ability in this respect, seems to be justified.

Nevertheless, the differences in red color vision may depend on subtle gene variations affecting the opsin gene. The most common variation at position 180 (A/S180), determining the existence of alanine or serine, causes a shift in λ_max_ absorption spectrum by about 3nm (557.5±0.4 nm [R(A180)]; vs 560.4±0.3 nm [R(S180)] [10]. Assuming that the Rayleigh test is performed using the diode 666 nm and that a relatively high bias in red cones absorption spectrum is observed at 666 nm, the observed small λ_max_ absorption shift may cause a relatively high change in opsin absorption at 666 nm. Thus, it must be analyzed if the postulated subtle protanomaly subjects possess in fact only the A/S180 genetic variant of the opsin protein and posses slightly weaker sensitivity at 666nm but not in remaining area of red cones spectrum.

The other parameter that could be related to the Rayleigh test parameters is the difference between the maximum absorption spectra for L and M cones since the spectra are similar and only about 30nm apart. Thus, even a tiny shift in the absorption spectra between L or M cones may contribute to a significant increase in distinguishing the hues and tones between red and green.

The results of this study confirmed that human eyes are highly sensitive to red-green colors (narrow R_W_). Red-green deficits are much more common in the population, especially in males, as this congenital defect is related to the X chromosome responsible for coding the L/M cones [24]. Acquired color vision disorders in the red and green area occur in cases of optic neuritis and multiple sclerosis [18]. Thus, the measurement of color vision sensitivity using an anomaloscope, which is a highly sensitive method, could be a useful tool for diagnosing and/or monitoring ocular pathologies. When this technique is to be applied as a diagnostic test, the authors suggest to use a wider reference range for the Rayleigh test during examinations.

### The Moreland test

The data presented for the Moreland test indicates very high interpersonal differences in the results. The central point of the equalization range (M_C_) is a parameter that describes the relative ability to see blue and green colors. A high value of this parameter may be interpreted as a relatively lower sensitivity to green as compared to blue. On the contrary, lower values suggest a lower sensitivity to blue color. Fig 2B shows the distributions of all the calculated parameters of the Moreland test. The highest variability is noticeable for the M_1_ parameter which varies broadly between 0 and 78. However, M_2_ varies to a significantly lower extent, ranging mainly between 60 and 100 for individual eyes. Also, only in one case the value of M_2_ was 40. The width of the color equalization range (M_w_) showed a considerable variation in individual eyes in the range between 8 and 95. High M_W_ value indicates a poor ability to distinguish between different shades and tones of green and blue. In the majority of the population, the parameter is usually higher than 60. Such individuals poorly distinguish the shades and tones of green and blue and perceive a wide range of green/blue diode proportions as the same color. When green light is added, only increased brightness is perceived but the perceived hue remained unchanged. However, certain individuals show a narrow range of equalization, which means that they are able to distinguish more shades of a color (e.g. see colors such as turquoise and aquamarine). The intersubject differences regarding green-blue area detection may result from different distributions of S-cones on their retinas. The S-cones are also less numerous than the L and M-cones and represent only 2% of the total number of cones in the central fovea [25]. Thus, it is extremely difficult to define a normal range for the Moreland test in order to make it a useful diagnostic tool. However, the issue seems important since the Moreland test could be used as an additional diagnostic tool to help identify groups of patients at the onset of losing crystalline lens transparency, for example due to cataract [25, 26] or as a result of retinal changes in glaucoma [13, 27] or diabetes [14, 28]. However, as this study has shown, the procedures defined for the Oculus HMC Anomaloscope MR as of today, do not enable satisfactory detection of color deficits in this area.

It is difficult to determine the reasons for high interpersonal variability in the Moreland test and it was also out of the scope of this work. The authors suggest three reasons for this phenomenon:

1. The density of S-cones in the retina is low and thus even small interpersonal variations in their density may cause significant differences in the Moreland test.
2. The S-cones are located mainly in the peripheral retina and therefore the width of the illuminated field of the anomaloscope (2deg) may be too small in order to correctly measure blue color deficits.
3. The design of the Moreland test is imperfect. The wavelength of the cyan diode (480nm) is very similar to the wavelength of the green diode (490nm). The difference may be too small to properly estimate blue-green color vision differences and may cause problems with color matching. In the opinion of the authors of this study, another problem seems to be the high intensity of the blue diode which dominates the color impression in the reference field.

Further studies are necessary in order to make the test more reliable and sensitive, possibly using additional sensitivity tests for hue brightness perception. This issue falls into the scope of future research planned by the authors.

### Correlations between the Rayleigh and the Moreland tests

The correlations between the Rayleigh and the Moreland parameters are most significant between R_1_/R_W_ and M_1_/M_W_/M_C_, respectively. R_2_ is also correlated with the Moreland parameters, however, the correlation is poorer than that between R_1_ and R_W_. Since M_C_ and M_W_ depend on M_1_ (as M_2_ is quite stable) and R_W_ depends on R_1_, the relation between R_1_ and M_1_ seems to be the most promising one for further analysis.

R_1_ is significantly correlated with the Moreland test parameters as it is highly related to green light sensitivity. Subjects with slightly poorer green detection (low R_1_) also showed a lower ability to distinguish the hues between blue and green (low M_1_, high M_W_). It should be pointed out that R_1_ increases together with the increment of M_1_ and such relation was most significant for high M_1_. High M_1_ group (M_1_≥40) exhibited good sensitivity to blue color as compared to green. This group also showed low M_W_, which indicates a good ability to distinguish between different hues and tones of green and blue. Also in the Rayleigh test, these subjects were able to see green well (high R_1_) and showed narrow R_W_, which denotes a good distinguishing of tones between green, yellow and red. It may be concluded that such individuals show a relatively better ability to see blue and green in comparison to red.

It has also been found that R_2_ was negatively correlated with M_1_ and positively with M_W_. It means that good red color vision (low R_2_) to some extent improves the ability to distinguish the hues and tones in the green/blue axis (narrow M_W_). This observation may depend on the L cones which have a broad absorption spectrum extending into the range of blue wavelengths. Stimulation of the L cones with blue and green light may provide additional information and improve the distinction between hues of blue and green.

According to a rival theory of color vision process [29], the perception of blue depends on the yellow-blue channel which relies on M and L cones. Therefore, both M_1_ and R_1_ in subjects with M_1_≥40 may be due to high sensitivity to green. However, there is a large group of individuals who show a low M_1_ (M_1_<40) with highly varied R_1_ values. These subjects have a relatively poorer ability to see blue. It may be concluded that the poorer ability to see blue does not influence the results of the Rayleigh test.

The last issue concerns the potential ability the blue diode, used in the Moreland test (430nm), to slightly stimulate the M and L cones. This ability may cause a moderation of the Moreland test results by stimulation of the red cones. The authors also consider this factor to be a probable cause of the specific pattern of the M1 vs R1 correlation presented and discussed in Fig 4. However, this hypothesis requires a deeper analysis.

### Limitation of the study and future directions

The authors have found differences in color perception between genders, however, the numbers of female and male participants were not equal. In future studies, it would be advisable to increase the number of males in order to investigate gender differences in color vision using the Moreland test.

Additionally, the R_2_ ≥ 50 group was the least numerous one, which could have influenced the statistics. It would be interesting to carry out tests in a larger group in order to find more subjects with high R_2_ and to try to correlate this parameter to certain genetic mutations. In future studies, it would also be interesting to check the differences between the eyes, especially in older individuals, as certain ocular pathologies might affect color vision sensitivity.

## Conclusions

The results of the present study show that the built-in Moreland test of the Oculus HMC Anomaloscope MR should not be used to diagnose blue color vision deficits. In the group of subjects who showed normal blue color vision in the HRR test, the majority had M_1_ values below 40 and in a broad range between 0 and 40. Due to the above, the test can falsely indicate deficits in blue color vision. However, the test in its present form is able to distinguish between individuals whose ability to perceive the blue color is very good from those whose blue light perception is poor. From this perspective, the method should not be used as a diagnostic test. However, it may be applied to monitor the deterioration in the perception of blue in subjects with good baseline blue vision who suffer from illnesses that may potentially deteriorate this ability (such as glaucoma, diabetes and cataract). However, further research is required in this area.

## Supporting information

S1 Appendix (DOCX)

S1 File (XLSX)
