# Supplementary material for: Variability of Rayleigh and Moreland test results using anomaloscope in young adults without color vision disorders
Source: PLoS One. 2021 May 21;16(5):e0251903. doi: 10.1371/journal.pone.0251903 (PMC8139452; doi:10.1371/journal.pone.0251903)
Supplement: S1 Appendix — (DOCX) [file pone.0251903.s001.docx]

**Appendix**

**HRR test**

The HRR test was performed monocularly in the room with intensive artificial light. If the person had problem at anyone level of the test, he/she was excluded from the further experiment. Next, the subject who fulfilled correctly HRR test, was send for optometric examination (visual acuity and refractive error measurements).

**Anomaloscope test**

This test was performed after the HRR and optometric examinations. HMC Anomaloscope MR type 47700 (Oculus) was used. The non-tested eye was covered with black patch. The order of eyes was counterbalanced. The ilumination in the room was low. The persons were adopted to the room ilumination by 10 minutes. Before the main test, the demo test was performed in order to explain the measurement procedure.

Both Rayleigh and Moreland tests were performed manually. Firstly, the Rayleigh test was carried out. The upper and lower limit has been found using the staircase method. The initial value of the reference field was 40 units. The measurement of higher limit (R_2_) preceeded lower limit test (R_1_). For R_2_, the reference field was increased by 5 up to reaching the lack of possibility of adjusting the reference and test field. Next, the actual range for R_2_ was divided by half in order to tighten the range of R_2_. This procedure was repeated up to reaching the measurement limit of the device equal to 0.5. Next, R_1_ was measured in simiar way. The R_1_ and R_2_ values were measured with precision 0.5. After the first eye examination, 2-3 minutes break was performed and the second eye was measured with Rayleigh test.

After the next 2-3 min break, the Moreland test started. The initial reference value was 50 units. The measurement of M_2_ preceeded M_1_. For M_2_, the reference value increased by 10 up to reaching the lack of possibility of adjusting the reference and test field. Next, the actual range for M_2_ was divided by half in order to tighten its range. The procedure was repeated up to reaching the precision of M_2_ equal to 1 unit. After one eye was measured, 2-3 minutes break was performed and the second eye was measured.
